# Efficacy and Safety of CT-Guided Patent Blue Injection to Localize Deep Pulmonary Nodules of the Thorax

**DOI:** 10.3390/medicina61061027

**Published:** 2025-05-31

**Authors:** Cheng-Hsun Lin, Tsai-Wang Huang, Hsian-He Hsu, Wen-Chiuan Tsai, Kai-Hsiung Ko

**Affiliations:** 1 Department of Radiology, Tri-Service General Hospital, National Defense Medical Center, Taipei 114, Taiwan; jason177623@gmail.com (C.-H.L.); hsianhe@yahoo.com.tw (H.-H.H.); 2 Division of Thoracic Surgery, Department of Surgery, Tri-Service General Hospital, National Defense Medical Center, Taipei 114, Taiwan; chi-wang@yahoo.com.tw; 3 Department of Pathology, Tri-Service General Hospital, National Defense Medical Center, Taipei 114, Taiwan; drtsaiwenchuan@mail2000.com.tw

**Keywords:** CT-guided localization, patent blue dye, deep pulmonary nodule, video-assisted thoracoscopic surgery

## Abstract

*Background and Objectives*: The needle path is crucial for preoperative localization of deep thoracic pulmonary nodules using CT-guided patent blue dye (PBD) injection. This study aimed to evaluate the efficacy and safety of four categorized needle approach paths tailored to the anatomical location of the nodules. *Materials and Methods*: We retrospectively evaluated data from 50 consecutive patients (50 deep pulmonary nodules), who underwent CT-guided localization with PBD injection, between November 2015 and May 2023 at our hospital. The nodules could be divided into four categories: (1) perifissural nodules, (2) paravertebral nodules, (3) paramediastinal nodules, and (4) deep parenchymal nodules, according to their location relative to the thoracic organs and the visceral pleura. Needle approach methods and needle pathway lengths were recorded. Clinical and radiological features, technical information, pathological results, and procedure-related complications were analyzed. *Results*: All 50 dyes were successfully identified by thoracoscopy and then resected without major complication. The mean nodule diameter and the nodular depth were 10.3 (range, 4.7–21.0) mm and 16.1 (range, 0.1–52.2) mm. The needle pathway length was 7.7 (range, 4.5–11.7) cm. The mean procedure time was 16 (range, 8–26) minutes. Asymptomatic pneumothorax developed in twenty-four patients (48.0%), and focal parenchymal hemorrhage occurred in four patients (8.0%) after localization. No patients required chest tube insertion or resuscitation. *Conclusions*: Strategic needle approach paths provide precise localization of deep thoracic pulmonary nodules with minimal complications. These methods offer a practical framework for improving thoracoscopic surgery in challenging deep thoracic cases.

## 1. Introduction

With the increasing prevalence of low-dose computed tomography (LDCT) for lung cancer screening, the detection of pulmonary nodules has significantly risen [1]. Among these, deep pulmonary nodules represent a unique challenge in video-assisted thoracoscopic surgeries (VATSs) due to their small size, ground-glass matrix appearance, and deep location relative to the pleural surface [2]. Accurate localization of these nodules prior to surgery is essential to ensure successful resection while minimizing the need for open thoracotomy, which is associated with increased morbidity [3]. Moreover, this challenge is further compounded for nodules situated deeply in the central thorax, where conventional localization methods often prove inadequate.

Several techniques have been developed for preoperative localization of pulmonary nodules. These include CT-guided hook-wire placement, microcoil localization, and injection of dyes, barium, or radiotracer [4,5,6,7,8,9,10,11,12,13,14,15]. While hook-wire techniques are widely adopted, they are associated with patient discomfort and a risk of dislodgement. They also have “blind areas” in deep thoracic locations, such as the mediastinum vicinity, areas near the interlobar fissures, and scapula-shadowed regions [3,16]. Microcoil placement has emerged as a safer alternative for these scenarios, but its reliance on intraoperative fluoroscopy adds complexity and radiation exposure. Disadvantages such as coil migration and air embolism have also been reported [3,6,7]. Dye injection techniques are simpler and cost-effective but have historically been limited by dye diffusion issues, particularly with methylene blue [3,8,9,12].

Patent blue dye (PBD) offers a potential solution to these challenges. Its precise marking capabilities, even in deep-seated nodules, and its long staining effect make it a promising tool for localization [11,17]. However, achieving successful localization for deeply situated nodules requires careful consideration of factors such as needle depth, which may be associated with complications like pneumothorax and pulmonary hemorrhage [18]. These complications are likely related to the extent of lung parenchyma penetration, emphasizing the need for strategic planning of the needle pathway. Additionally, Tsai et al. highlighted the importance of combining superficial marking and deep marking for successful localization of deeply situated pulmonary nodules [19]. Despite these advancements, the utility of patent blue injections specifically for deep thoracic nodules remains under-researched.

To address this gap, we categorized four scenarios based on anatomical locations, applied corresponding needle approach paths, and injected the dye from the nodule to the subpleural area. This retrospective study aims to evaluate the efficacy and safety of these methods.

## 2. Materials and Methods

**Figure 1:**
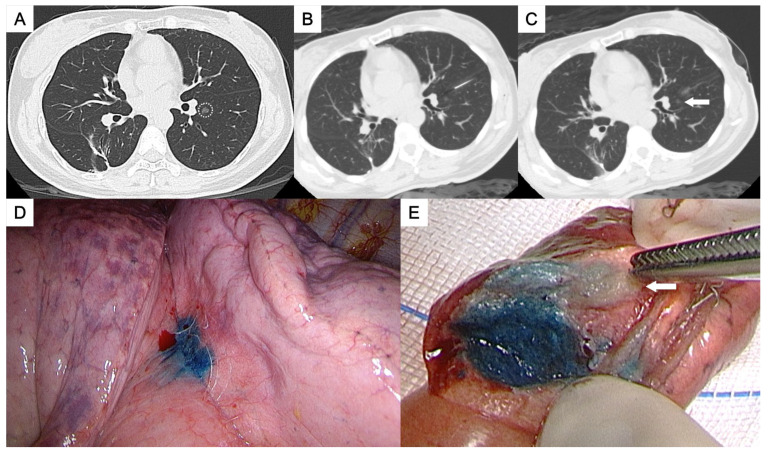
(**A**) A ground-glass nodule in the left lower lobe near the interlobar fissure (circle). (**B**,**C**) Patent blue dye was injected into the subpleural area adjacent to the nodule (arrow). (**D**) Successful localization with a visibly stained area on the visceral pleura. (**E**) The tumor (arrow) was clearly identified in the resected specimen. The pathological result was adenocarcinoma.

**Figure 2:**
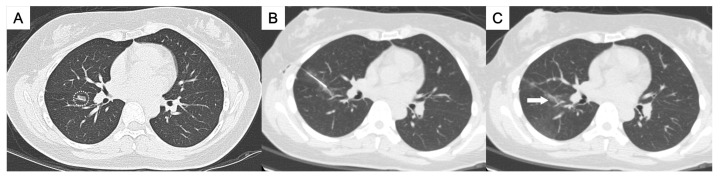
(**A**) A ground-glass nodule in the right lower lobe near the interlobar fissure (circle). (**B**,**C**) A transfissural puncture was performed, followed by patent blue dye injection into the subpleural area near the nodule (arrow). The pathological result was adenocarcinoma.

**Figure 3:**
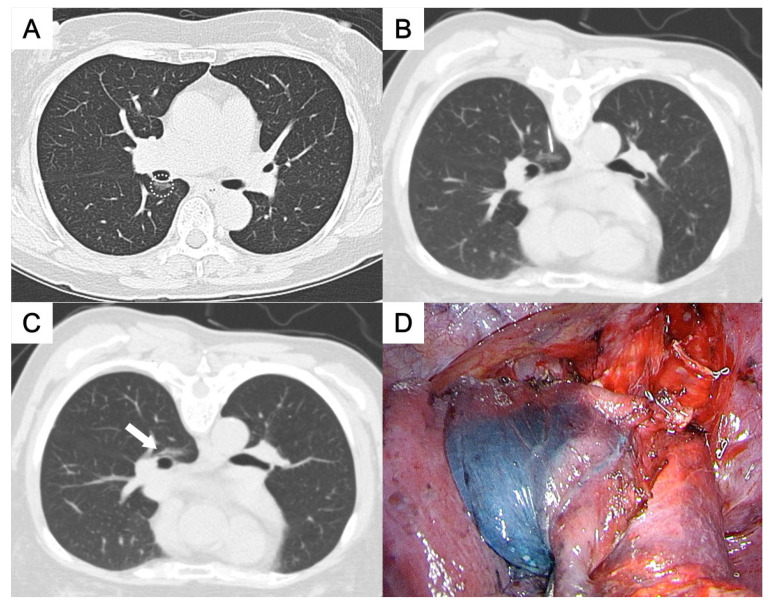
(**A**) A ground-glass nodule in the paravertebral region of the right lower lobe (circle). (**B**,**C**) A paraspinal approach was used, with patent blue dye injected near the nodule (arrow) and along the needle pathway to the subpleural area. (**D**) The stained area was clearly visible on the visceral pleura. The pathological result was adenocarcinoma.

**Figure 4:**
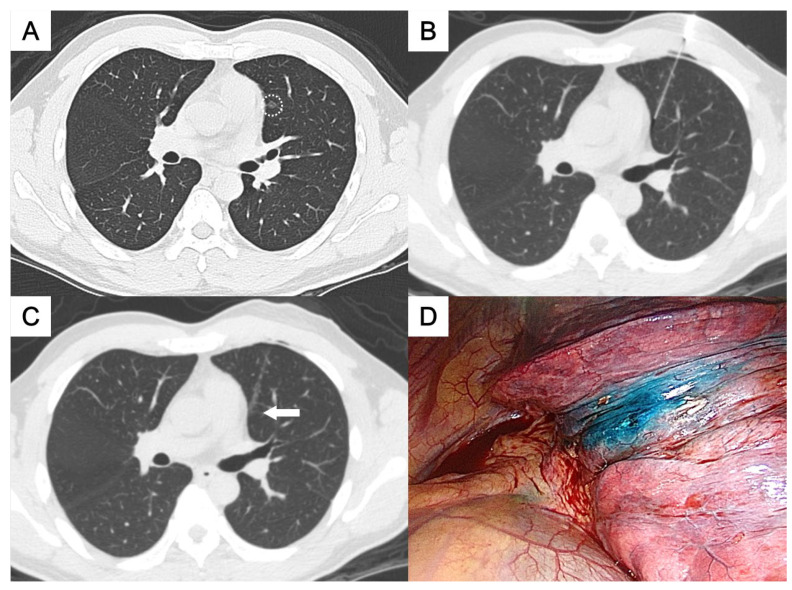
(**A**) A ground-glass nodule in the left upper lobe near the mediastinum (circle). (**B**,**C**) Patent blue dye was injected into the subpleural area medial to the nodule (arrow). (**D**) The stained area was well visualized on the visceral pleura. The pathological result was minimally invasive adenocarcinoma.

**Figure 5:**
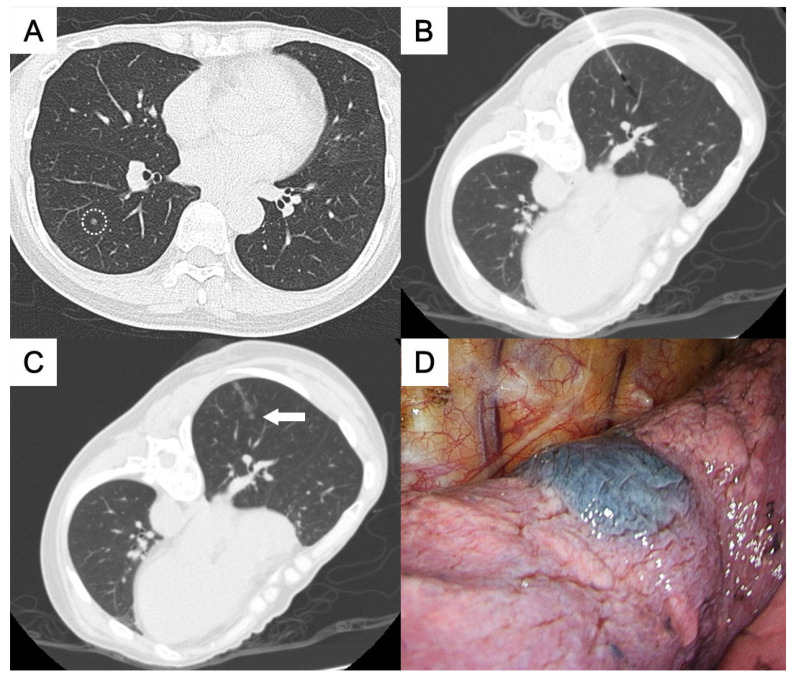
(**A**) A deeply located ground-glass nodule in the right lower lobe (circle). (**B**,**C**) Patent blue dye was injected near the nodule (arrow) and along the needle pathway to the subpleural area. (**D**) Video-assisted thoracoscopy confirmed the dye marking on the visceral pleura. The pathological result was adenocarcinoma.

This study was approved by the Institutional Review Board for human research, with a waiver of informed consent. During the period between November 2015 and May 2023, 50 consecutive patients who underwent CT-guided localization with the injection of PBD were enrolled, all of whom had pulmonary nodules located deep within the central thorax. The definition of “deep pulmonary nodules of the thorax” and their categorization according to the nodule localization relative to the thoracic organs and the visceral pleura, which required different needle approach pathways, were as follows:

- Perifissural nodules: nodules situated adjacent to the interlobar fissure, typically in the mid thorax (Figure 1 and Figure 2);
- Paravertebral nodules: nodules located near the lateral border of the thoracic spine, typically in the posterior thorax (Figure 3);
- Paramediastinal nodules: nodules located adjacent to the mediastinal pleura in close proximity to mediastinal structures such as the mediastinal vessels or heart border, typically in the anterior thorax (Figure 4);
- Deep parenchymal nodules: nodules situated within the central portion of a pulmonary lobe, remote from any pleural surface including the fissures (Figure 5).

The criteria for preoperative localization were as follows: (1) nodules measuring less than 3 cm in diameter, (2) nodules not in direct contact with the visceral pleura, and (3) nodules deemed challenging for intraoperative identification by surgeons. Surgical intervention was indicated for nodules with increasing size or a growing solid part. Additionally, some patients with stable subsolid nodules (solid part < 5 mm) opted for surgery due to concerns about malignancy. For each patient, clinical parameters were recorded, including age, sex, smoking status, body mass index, and the type of surgical procedure. CT findings for each nodule were analyzed, including (1) nodule size, (2) pulmonary lobe involvement, and (3) attenuation. Several technical aspects of the localization procedure were also recorded, such as the (1) nodule localization category, (2) nodule depth (the distance between the nodule and the nearest pleura), (3) the needle pathway length (from the skin insertion to the tip of the needle), (4) the distance between the nodule and the dye, and (5) the procedure duration. The time interval between localization and surgery, along with any post-localization complications, was recorded. Complications included pneumothorax (asymptomatic or symptomatic, requiring preoperative intervention), parenchymal hemorrhage, hemothorax, hemoptysis, and allergic reactions to PBD. All CT scans were independently reviewed by two chest radiologists (H.H.H. and K.H.K., with 32 and 17 years of experience). Discrepancies were resolved through discussion.

### 2.1. CT-Guided PBD Localization Procedure

One experienced chest radiologist (K.H.K.) conducted all the localization procedures. Detailed information regarding the main parameter settings for the CT device [64-detector row scanner (Brilliance; Philips Medical Systems, Cleveland, OH, USA)] and the CT-guided PBD (patent blue V 2.5%; Guerbet, Aulnay-sous-Bois, France) injection had been described in our previous study [17]. In brief, all procedures were performed using a low-dose non-contrast CT protocol for planning. After local anesthesia, a 22-gauge Chiba needle was inserted under intermittent CT guidance without fluoroscopy. Once the needle tip was confirmed to be in the appropriate position close to the target nodule for dye injection, PBD was incrementally injected during controlled needle retraction. The needle approach method and specific injection strategies for each category of deep thoracic nodules were as follows:

- For the perifissural nodules (Figure 1), we introduced the needle using the shortest path from the chest wall to the nodule and injected the PBD into the subpleural area. There is an alternative transfissural approach (Figure 2) to avoid pulmonary vessels, scapula, and ribs, with dye retention in the subpleural area.
- For the paravertebral nodules (Figure 3), we introduced the needle with the paraspinal approach, from the posterior chest wall with the tip placed within 1 cm of the nodule. PBD was then injected while withdrawing the needle, leaving dye along the needle pathway until reaching the subpleural area.
- For the paramediastinal nodules (Figure 4), we approached the nodule using the shortest way, from the chest wall to the nodule, with the needle tip placed within 1 cm on the relative medial side of the nodule. We then injected the PBD, with the dye retention in the subpleural area.
- For the deep parenchymal nodules (Figure 5), the needle pathway followed the shortest path from the chest wall to the nodule, while avoiding bony structures. After confirming that the needle tip was placed within 1 cm of the nodule, the PBD was injected, retracting the needle until reaching the subpleural area. The dye was then deposited along the needle pathway.

Following localization, patients were sent back to the ward while awaiting transfer to the operating room. The procedure duration was measured from the first to the last CT scan.

### 2.2. Video-Assisted Thoracoscopic Surgery (VATS) After Localization

VATS was performed on the same day or the following day after CT-guided preoperative localization, depending on the surgeon’s schedule and operating room availability. Successful localization was defined as the presence of a dyed area on the visceral pleura, facilitating wedge resection with complete nodule removal and negative pathological margins. The resected specimens were sent for frozen section analysis. Limited wedge resection or segmentectomy was performed for benign lesions, particularly in patients with limited cardiopulmonary reserve or for nodules smaller than 2 cm with a predominant ground-glass matrix appearance [20]. In cases of malignancy, anatomic resection and mediastinal lymph node dissection were performed. Surgical approaches were adjusted based on the surgeon’s judgment and the intraoperative circumstances. Technical failure was defined as the lack of visible dye marking on the visceral pleura.

### 2.3. Statistical Analysis

Categorical variables are reported as numbers and percentages, while numerical variables are presented as means or medians with ranges. The chi-square test was used for categorical variables, and the Mann–Whitney test was applied for non-normally distributed continuous variables. Multivariate logistic regression analysis was performed to identify risk factors for post-localization pneumothorax. A *p*-value < 0.05 was considered statistically significant. All analyses were conducted using SPSS software (version 21.0; SPSS, Chicago, IL, USA).

## 3. Results

### 3.1. Clinical and Radiological Features

The characteristics of the 50 enrolled patients with 50 indeterminate pulmonary nodules are summarized in Table 1. The patients were predominantly middle aged (mean = 57.4 years), female (n = 39; 78.0%), and non-smokers (n = 39; 78.0%). The BMI ranged from 17.4 to 29.7 (mean = 23.0), covering underweight, normal, and mildly obese individuals, with no cases falling into the moderate or severe obesity categories. The mean size of the nodules was 10.3 mm (range, 4.7–21.0) and most had a pure ground-glass appearance (n = 40; 80.0%).

### 3.2. Localization Parameters, Complications, and Surgical Results

The localization parameters, associated complications, and surgical results are shown in Table 2. The nodules could be categorized as (1) perifissural nodules (n = 13; 26.0%), (2) paravertebral nodules (n = 20, 40.0%), (3) paramediastinal nodules (n = 4; 8.0%), and (4) deep parenchymal nodules (n = 13, 26.0%). The mean distance between the nodule and the nearest pleural surface was 16.1 mm (range, 0.1–52.2 mm). The mean needle pathway from the chest wall was 7.7 cm (range, 4.5–11.7 cm). Among the thirteen perifissural nodules, eleven were approached using the transfissural path, while the remaining two were localized via an alternative path to avoid pulmonary vessels. The median of the distance between the nodule and the dye, measured with a CT scan after dye injection, was 0.6 mm (mean, 1.6 mm; range: 0–13.5 mm). The mean procedure time was 16 min (range, 8–26). The median of time interval from dye injection to the operation was 139 min (mean, 270; range, 57–2641). A longer time interval was due to a changed operation schedule. All 50 dyes could be identified on the pleural surface by thoracoscopy and were resected mostly by wedge resection (n = 25, 50.0%).

For the localization related complications, asymptomatic pneumothorax developed in twenty-four patients (48.0%), and focal parenchymal hemorrhage was identified in four patients (8.0%). Multivariate logistic regression analysis identified no specific risk factors for procedure-related pneumothorax (Table 3).

### 3.3. Pathological Reports

The majority of nodules (n = 48, 96.0%) were malignancies, including invasive adenocarcinoma, minimally invasive adenocarcinoma (MIA), adenocarcinoma in situ (AIS), squamous cell carcinoma, and atypical adenomatous hyperplasia (AAH) (Table 4). All surgical margins were negative for tumor involvement.

## 4. Discussion

This retrospective study aimed to assess the efficacy and safety of CT-guided patent blue dye injection for localizing deep pulmonary nodules in the thorax. Our focus was on developing effective needle path planning to ensure accurate marking on the visceral pleura while minimizing complications, such as pneumothorax and pulmonary hemorrhage, which may be linked to excess lung parenchyma penetration. By categorizing nodules into four anatomical scenarios—(1) perifissural, (2) paravertebral, (3) paramediastinal, and (4) deep parenchymal—and adopting tailored needle approaches, the results showed a high success rate (100%), a relatively short procedure time (mean: 16 min), and minimal complications.

Regarding marking deep central thoracic locations, two core objectives must be achieved: creating a visible marking on the nearest visceral pleura and accurately localizing the nodule within the parenchyma to guide surgeons in obtaining an adequate surgical margin while preserving functional lung tissue. For perifissural nodules, dye injection in the subpleural area near the nodule, with or without transfissural puncture, provides a precise stained area on the fissure. For paravertebral nodules, a paraspinal approach allows the shortest path to the nodule in the prone position, with dye injected along the needle tract to create a clear marking on the posterior visceral pleura. Similarly, for paramediastinal nodules, dye injection into the subpleural area closest to the mediastinum provides precise marking relative to the mediastinal-side visceral pleura. For deep parenchymal nodules, dye is injected along the needle path to create a blue tract extending to the subpleural area, enabling visible marking on the nearest visceral pleura while indicating the lesion’s location within the parenchyma. In all cases, the needle tip was placed within 1.0 cm of the nodule, ensuring effective dye diffusion to the lesion site, resulting in a close distance between the nodule and the dye (median: 0.6 mm; mean: 1.6 mm; range: 0–13.5 mm). The results demonstrate the reliability of this core concept, aligning with prior results on deep parenchymal nodules by Tsai et al. [19].

In terms of complications, pneumothorax and parenchymal hemorrhage are the most common. In our study, pneumothorax occurred in 48% of patients, all of which were asymptomatic and required no further intervention. This rate is slightly higher than that reported by Lin et al., who observed pneumothorax in 29.4% of patients undergoing CT-guided patent blue dye (PBD) localization [11], and Tsai et al., who reported a pneumothorax rate of 37.0% specifically in cases involving deep pulmonary parenchymal nodules [19]. This discrepancy may be attributable to our focus on deep thoracic nodules, which required a deeper needle pathway (mean 7.7 cm; range 4.5–11.7 cm) and included transfissural punctures in 22% of cases. Parenchymal hemorrhage was noted in 8% of our cases, all of which were focal and self-limiting. Regarding allergic reactions, none occurred in our cohort. PBD is commonly used in lymphangiography and sentinel node mapping, where allergic effects—ranging from rash to anaphylaxis—have been reported. According to a large meta-analysis, the overall anaphylaxis rate for patent blue dye in sentinel lymph node procedures is approximately 0.05%. Moreover, low dye volumes (<2 mL) have been associated with lower anaphylaxis rates [21]. Although the allergic risk in pulmonary localization remains uncertain, the low injection volume used in our protocol may have contributed to the absence of such events. Likewise, deep dye migration was not observed in our series, possibly due to careful injection technique and limited dye volume, which may help mitigate this risk. Other potential complications—such as hemothorax, hemoptysis, systemic air embolism, and inadvertent vascular injury—were not encountered in our series. Nevertheless, these have been reported in CT-guided thoracic procedures [7] and should be recognized as rare but possible risks. Our results indicate that with careful planning and technique, PBD localization can be performed with a favorable safety profile, even in challenging cases involving deep thoracic nodules.

When comparing localization techniques for deep thoracic nodules, the CT-guided patent blue dye (PBD) injection method demonstrates several practical advantages over hook-wire, microcoil, and dual localization methods. Hook-wire localization, while effective for peripheral nodules, is often associated with dislodgement, patient discomfort, and limited utility in challenging areas such as the mediastinum or interlobar fissures [16]. Microcoil localization provides a reliable depth marker but requires intraoperative fluoroscopy, introducing additional procedural complexity and radiation exposure. Dual localization techniques, combining microcoils and PBD [22], offer both surface and depth marking but involve longer procedural times (mean: 39 min), which may not be feasible in all settings. Patent blue dye injection, combined with a tailored needle approach strategy, offers a simple and straightforward protocol. While our approach does not provide specific markers for deep safe margins as some dual localization techniques do, this study indicates that it provides sufficient information on nodule location and depth for surgeons to achieve complete resection. However, this study did not directly compare the efficacy or complication profiles of PBD injection with other localization techniques. Future studies incorporating a prospective, randomized design with comparator arms would enhance the clinical utility of the findings.

This study has several limitations. It is a single-center, retrospective analysis with a relatively small sample size, which may limit the generalizability of our findings. In addition, the limited number of outcome events relative to the number of variables included in the multivariate analysis—such as age, BMI, and other potential risk factors—raises concerns about possible model overfitting. Moreover, the statistical power may have been insufficient to detect small-to-moderate associations. Although our method effectively localized deep thoracic nodules, its applicability to cases involving multiple nodules or nodules located outside the four anatomical scenarios warrants further investigation. Future research with larger, multi-center cohorts is needed to validate these results and explore the potential for broader applications.

## 5. Conclusions

The findings of this study demonstrated that the four categorized needle approach paths enabled precise and reliable PBD localization of deep thoracic pulmonary nodules for VATS with minimal complications. By providing a simple and effective framework, this method may serve as a valuable reference for less experienced operators, offering practical guidance to enhance confidence and consistency in such challenging cases.

## Figures and Tables

**Table 1:** Clinical and radiological features of 50 nodules in 50 patients.

	Value N (%) or Mean (Range)
**Age (years), Mean (range)**	57.4 (32–74)
**Sex**	
Male	11 (22.0%)
Female	39 (78.0%)
**Smoking status**	
Never	39 (78.0%)
Current or former	11 (22.0%)
**BMI, Mean (range)**	23 (17.4–29.7)
**Nodular size (mm), Mean (range)**	10.3 (4.7–21.0)
**Pulmonary lobe**	
RUL	16 (32.0%)
RML	1 (2.0%)
RLL	11 (22.0%)
LUL	16 (32.0%)
LLL	6 (12.0%)
**Nodule attenuation**	
Ground-glass opacity	40 (80.0%)
Part solid	4 (8.0%)
Solid	6 (12.0%)

Abbreviations: BMI, body mass index; RUL, right upper lobe; RML, right middle lobe; RLL, right lower lobe; LUL, left upper lobe; LLL, left lower lobe.

**Table 2:** Localization parameters, complications, and surgical results.

	N (%) or Mean (Range)
**Nodule localization category**	
Perifissural nodules	13 (26.0%)
Paravertebral nodules	20 (40%)
Paramediastinal nodules	4 (8%)
Deep parenchymal nodules	13 (26.0%)
**Transfissural puncture**	11 (22.0%)
**Nodular depth (mm)**	16.1 (0.1–52.2)
**Needle pathway length (cm)**	7.7 (4.5–11.7)
**Distance between the nodule and the dye (mm)**	Median: 0.6; Mean: 1.6; Range (0–13.5)
**Procedure time (minute)**	16 (8–26)
**Time interval from localization to surgery (minute)**	Median: 139; Mean: 270; Range (57–2641)
**Complications**	
Pneumothorax	24 (48.0%)
Asymptomatic	24 (48.0%)
Symptomatic	0 (0.0%)
Focal parenchymal hemorrhage	4 (8.0%)
Hemothorax	0 (0.0%)
Hemoptysis	0 (0.0%)
Allergic reaction	0 (0.0%)
Deep dye migration	0 (0.0%)
Systemic air embolism	0 (0.0%)
Inadvertent vascular puncture	0 (0.0%)
**Failed marking on the visceral pleura**	0 (0.0%)
**Surgical procedure**	
Wedge resection	25 (50.0%)
Segmentectomy	19 (38.0%)
Lobectomy	6 (12.0%)

**Table 3:** Risk factors for procedure-related pneumothorax.

	OR (95% CI)	*p* Value
Age	1.03 (0.95–1.13)	0.439
Smoking status	1.89 (0.23–15.77)	0.555
BMI	0.88 (0.67–1.12)	0.319
Nodular size (mm)	1.21 (0.95–1.54)	0.131
Pulmonary lobes	1.32 (0.77–2.29)	0.314
Attenuation of nodule	1.48 (0.40–5.48)	0.558
Localization type	1.59 (0.49–5.19)	0.439
Transfissural puncture	2.89 (0.31–26.59)	0.350
Nodular depth (mm)	0.93 (0.86–1.01)	0.094
Needle pathway length (cm)	0.88 (0.51–1.49)	0.626

**Table 4:** Pathological results.

	N (%)
**Malignant and premalignant lesions**	48 (96.0%)
Invasive adenocarcinoma	35 (70.0%)
MIA	6 (12.0%)
AIS	4 (8.0%)
Squamous cell carcinoma	1 (2.0%)
AAH	2 (4.0%)
Metastasis	0 (0%)
**Benign lesions**	
Chronic granulomatous inflammation	2 (4%)
Fibrosis	0 (0%)
Intrapulmonary lymph node	0 (0%)

Abbreviations: MIA, minimally invasive adenocarcinoma; AIS, adenocarcinoma in situ; AAH, atypical adenomatous hyperplasia.

## Data Availability

The raw data supporting the conclusions of this article will be made available by the authors, without undue reservation.

## Abbreviations

The following abbreviations are used in this manuscript:

CT	Computer tomography
PBD	Patent blue dye
VATS	Video-assisted thoracoscopic surgery
BMI	Body mass index
RUL	Right upper lobe
RML	Right middle lobe
RLL	Right lower lobe
LUL	Left upper lobe
LLL	Left lower lobe
MIA	Minimally invasive adenocarcinoma
AIS	Adenocarcinoma in situ
AAH	Atypical adenomatous hyperplasia
